# Retraction: Abnormally expressed JunB transactivated by IL-6/STAT3 signaling promotes uveal melanoma aggressiveness via epithelial-mesenchymal transition

**DOI:** 10.1042/BSR-2018-0532_RET

**Published:** 2023-05-23

**Keywords:** EMT, interleukin 6, JunB, STAT3, uveal melanoma

This article is being retracted from *Bioscience Reports* at the request of the authors following receipt of a notification from a reader, alerting the Editorial Office to multiple similarities between the images within this paper and with those in papers of unrelated authors. Similarities noted between images are described below:

- Figure 1A Transwell IL6/C918 panel and the Figure 1D Fra2 T98G panel from Luo et al. 2018 (doi: 10.1016/j.prp.2018.09.017)
- Figure 5C EV siSTAT3 panel and the Figure 4B U251 miR-124-3pmimics/EV panel from Luo et al. 2018 (doi: 10.1016/j.prp.2018.09.017)
- Figure 5C EV siSTAT3 panel and the Figure 3F bottom +/+ Transwell U251 cells panel from Zhang et al. 2017 (doi: 10.1042/BSR20160643)
- Figure 5C EV siSTAT3 panel and the Figure 2A +-+ U87MG panel from Su et al. 2019 (doi: 10.1186/s11658-019-0149-x)
- Figure 5C JunB siSTAT3 panel and the Figure 2B ++- A172 panel from Su et al. 2019 (doi: 10.1186/s11658-019-0149-x)
- Figure 1B Transwell IL6/OCM1A panel and the Figure 2C Hypoxia - panel from Fei et al. 2018 (doi: 10.1186/s11658-018-0100-6)
- Figure 6F MMP14 western blot bands and the Figure 3E p-STAT3 bands from Liang et al. 2020 (doi: 10.1042/BSR20193572)

The Editorial Office also identified various duplications within the paper:

- Between the Figure 2G TJP2 western blots (after being horizontally flipped) and the Figure 4A p-STAT3 C(18 and IL6/C918 bands
- Between the Figure 2G TJP2 western blots (after being horizontally flipped) and the Figure 5A TJP1 EV bands
- Between the Figure 3F TJP1 siCtrl bands and the first three Figure F5 STAT3 bands (after a 180-degree rotation)
- Between the Figure 4A p-STAT3 bands and the first three Figure 5 TJP1 bands

The authors are unable to correct the article and wish to retract the article. The Editor-in-Chief and Editorial Board agree with the retraction.

